# Supplementary material for: Arginine Methylation of hnRNPK Inhibits the DDX3-hnRNPK Interaction to Play an Anti-Apoptosis Role in Osteosarcoma Cells
Source: Int J Mol Sci. 2021 Sep 9;22(18):9764. doi: 10.3390/ijms22189764 (PMC8469703; doi:10.3390/ijms22189764)
Supplement: Supplementary file 1 [file ijms-22-09764-s001.zip › ijms-1348555-supplementary.pdf]

Arginine methylation of hnRNPK inhibits the DDX3-hnRNPK interaction to play an anti-apoptosis role in osteosarcoma cells

Chiao-Che Chen, [#,1] Jen-Hao Yang, [#,1] Shu-Ling Fu,[2] Wey-Jinq Lin,[3] and Chao-Hsiung Lin[1,3,4*]

[1] Department of Life Sciences and Institute of Genome Sciences, National Yang Ming Chiao Tung University, Taipei 112, Taiwan

[2] Institute of Traditional Medicine, National Yang-Ming Chiao Tung University, Taipei 112, Taiwan

[3] Institute of Biopharmaceutical Sciences, National Yang Ming Chiao Tung University, Taipei 112, Taiwan

[4] Proteomics Research Center, National Yang Ming Chiao Tung University, Taipei 112, Taiwan

Supporting information

Supplemental Figures

Figure S1. U2OS-2RK cells exhibited more apoptosis than U2OS-WT cells under DNA damage.

Figure S2. The protein level of JUND was slightly increased upon DNA damage in U2OS-2RK cells but not in U2OS-WT cells.

Figure S3. Another DDX3 inhibitor, AVN A, did not obviously increase the cell apoptosis level or enhance the hnRNPK-DDX3 interaction during DNA damage.

Figure S4. RK-33 enhanced DNA damage-induced apoptosis in U2OS cells.

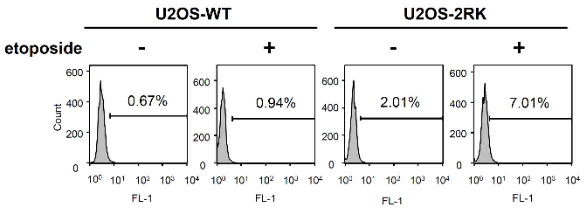

Figure S1. U2OS-2RK cells exhibited more apoptosis than U2OS-WT cells under DNA damage. U2OS-WT and U2OS-2RK cells were treated with etoposide (50 μM) for 12 h. Cells were collected and analyzed by TUNEL assay to determine the degree of apoptosis using FACS.

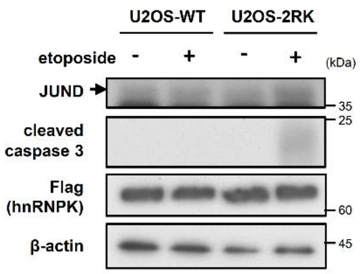

Figure S2. The protein level of JUND was slightly increased upon DNA damage in U2OS-2RK cells but not in U2OS-WT cells. U2OS-WT and U2OS-2RK (R296/299K) cells were treated with etoposide (50 μM) for 12 h. The resulting cells were collected and analyzed to determine the protein levels of active caspase-3 and JUND.

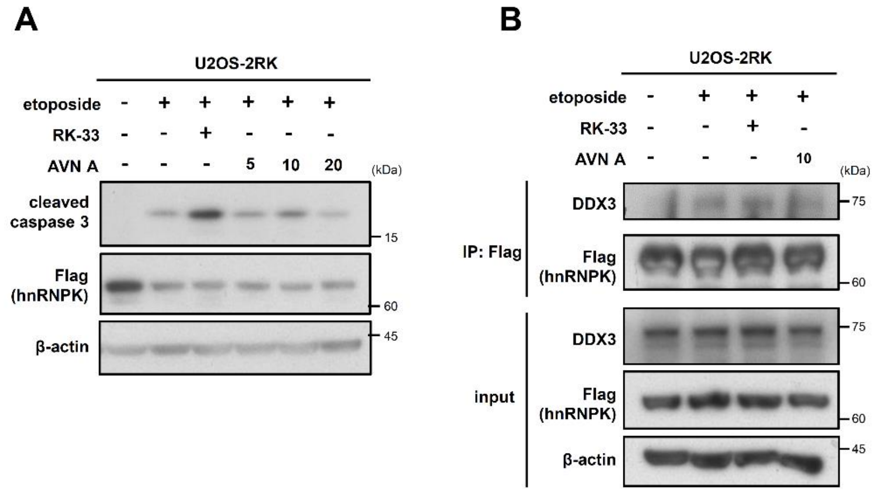

Figure S3. Another DDX3 inhibitor, AVN A, did not obviously increase the cell apoptosis level or enhance the hnRNPK-DDX3 interaction during DNA damage. (A) U2OS-2RK cells were treated with etoposide only (50 μM), etoposide together with RK-33 (5 μM), or AVN A (5, 10 and 20 μM) for 12 h. Cell lysates were collected and analyzed for the expression levels of active caspase-3. **(B)** Under the same treatments described above, cell lysates were collected at 4 h and the interaction between hnRNPK and DDX3 was assessed.

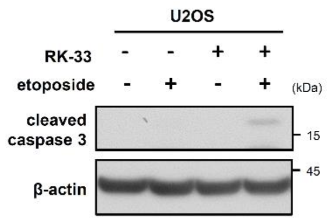

Figure S4. RK-33 enhanced DNA damage-induced apoptosis in U2OS cells. U2OS cells were treated with etoposide only (50 μM), RK-33 only (5 μM) or etoposide/RK-33 together for 12 h. Cell lysates were collected to determine the protein levels of active caspase-3.
